# Bioinspired metal complexes as electrocatalysts for hydrogen evolution: a systematic review

**DOI:** 10.1039/d6ra02934e

**Published:** 2026-07-06

**Authors:** José Renato Gomes Lopes, Larissa Chimilouski, Fernando Roberto Xavier, Carla Dalmolin

**Affiliations:** a Programa de pós-graduação em Química Aplicada, Universidade do Estado de Santa Catarina (Udesc) Joinville Brazil carla.dalmolin@udesc.br

## Abstract

The expanding environmental impact resulting from our dependence on fossil fuels is driving the search for sustainable alternatives for energy production. In this context, the hydrogen evolution reaction (HER), especially through the electrolysis of water, has emerged as a promising route for obtaining green hydrogen. This study presents a systematic review, according to the PRISMA guidelines, on electrocatalysts based on bioinspired metal complexes applied to HER. The search was carried out in the Web of Science database, resulting in a detailed analysis of 40 articles. Structural aspects of the catalysts, experimental conditions, electrochemical techniques and performance parameters such as overpotential, turnover frequency (TOF), faradaic efficiency and stability were investigated. The bioinspired complexes analysed include mononuclear, heterobimetallic and hybrid architectures, employing metals such as Co, Fe, Ni, Mo and W, with various ligands and supports. The results indicate that specific structural combinations can confer high catalytic activity, even with low overpotentials (<200 mV), and stability of more than 100 hours of continuous operation. The conclusion is that the engineering of bioinspired metal complexes represents a promising strategy in electrochemical catalysis for HER, but it requires integrated and multidisciplinary approaches, with greater methodological standardization and advances in the characterization of stability and selectivity under realistic conditions.

## Abstract 2

Active groups of hydrogenase enzymes can be mimicked by metal complexes for the reduction of H_2_.

## Graphical abstract

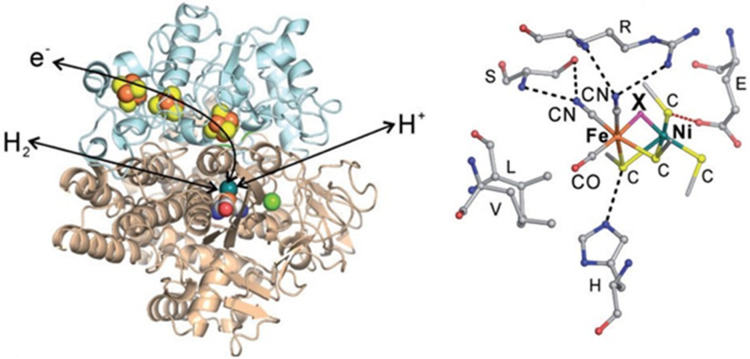

## 1. Introduction

Since the beginning of the 19th century, global energy consumption, driven by the industrial revolution, has grown exponentially, posing a challenge for sustainable development. At present, the most explored energy sources are fossil fuels such as coal, oil and natural gas, whose production is expected to double by 2030, with consequent socio-political and environmental impacts.^1^ This accelerated use of exhaustible resources is causing their extinction, as well as increasing the levels of CO_2_ emissions, resulting from their combustion, into the atmosphere. With such high levels of CO_2_ emissions, climate change is inevitable, causing damage to the less fortunate, such as the 3.3 billion people exposed to climate risks, and losses of around 7.9 trillion dollars for the world economy.^2,3^ Therefore, scientific and technological investments continue to be made to advance us towards the use of renewable and sustainable energy reserves.

Energy generation methods that use more easily-available resources with a lower environmental impact are finding space in this scenario, with strategies ranging from solar and wind to electrochemical energy, among others. From this variety of renewable energy resources, electrochemical energy stands out due to its storage and transportation capacity using different types of fuel cells.^4^ In general, this type of equipment works by converting the potential energy of an electrochemical reaction with a fuel. Thus, molecular hydrogen (H_2_) plays a role as an efficient fuel due to its high energy density and abundance compared to other elements.^5^ However, obtaining H_2_ takes different routes with possible limitations and challenges.

Molecular hydrogen, as an efficient carbon-neutral fuel, has known production routes with varying sustainability levels, *i.e.*, with different colour classifications^6^ in relation to the greenness of its synthesis. Synthetic routes that involve CO_2_ emissions, such as hydrocarbon or biomass reforming, are not considered sustainably viable. Also, the use of finite resources, such as nuclear energy, should be avoided. On the other hand, Hydrogen Evolution Reaction (HER) by water electrolysis is considered an environmentally friendly synthesis.^7,8^ In HER, green H_2_ is produced through the water splitting reaction; but, because its thermodynamic and kinetic barriers, studies are concentrated to find efficient electrocatalysts.

The catalytic efficiency of platinum and other noble metal electrocatalysts for HER is already well known experimentally. However, these metals are not easily available in nature, which raises their cost and renders them inaccessible to large production scales, also pushing the importance of catalysis using hydrogenase enzymes.^9,10^ These enzyme structures, which enable HER to be catalysed at a low overpotential, are inspiration for projects aimed to mimic efficient, low-cost electrocatalysts for H_2_ production.

**Fig. 1:**
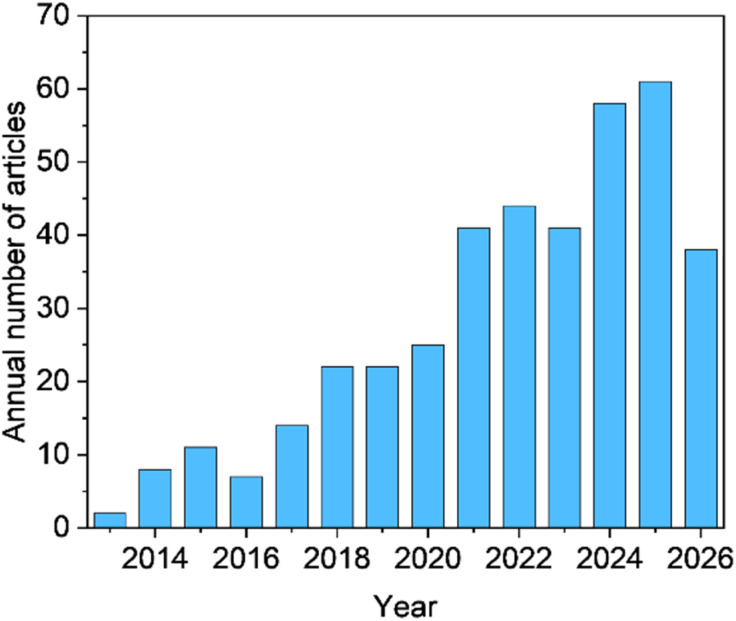
Number of publications per year for the search results in the Scopus database for the keywords “*electrocatalyst*”, “*HER*” and “*bioinspired*”.

A search was conducted in the Scopus database using the combination of keywords “*electrocatalyst*”, “*HER*”, and “*bioinspired*” reveals a significant growth and continuous scientific interest in this field over the last decade (Fig. 1). Between 2013 and 2023, a steady upward trend in the number of annual publications is noticeable, which culminated in a sharp increase from 2021 onwards, reaching its production peak in 2024 and 2025 with approximately 58 and 61 published articles, respectively. This recent production reflects the consolidation of bioinspired catalysts as one of the most promising frontiers for the global energy transition, driven by the urgent need to mimic the high efficiency of natural metalloenzymes (hydrogenases) in artificial and sustainable systems.

**Fig. 2:**
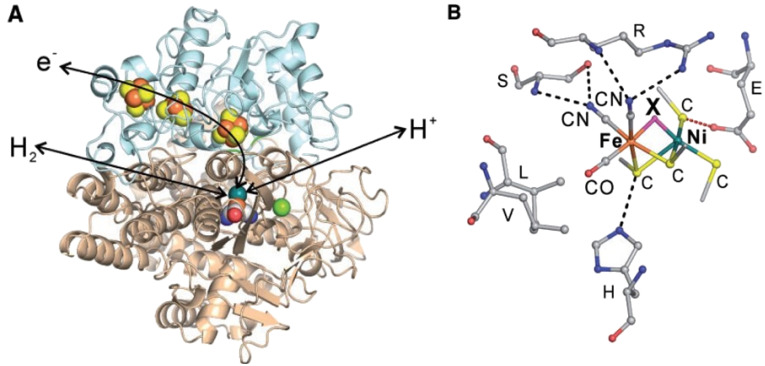
(A) The small and the large subunits are shown in light blue and light brown, respectively. The electron transfer pathway, and one of the proton transfer and gas access pathways are indicated by arrows. (B) Ball and stick representation of the Ni–Fe active site of the standard [NiFe] hydrogenase. The marked position (X) indicates the third bridging ligand, which changes during catalysis. Hydrogen bonds to the active site ligands are shown (dotted lines) and a possible proton transfer path is indicated by the dotted red line. This figure has been adapted from ref. 11 with permission from Ogata *et al.*, copyright 2016.

Hydrogenases are metalloenzymes classified according to the metal composition of their active sites into three major classes: [NiFe]-hydrogenases, [FeFe]-hydrogenases, and [Fe]-hydrogenases. These enzymes catalyze the conversion of molecular hydrogen into protons and electrons through a heterolytic cleavage mechanism.^11,12^ Hydrogenases are widely distributed among microorganisms and are found in archaea, bacteria, and some eukaryotes. In general, these enzymes contain metal centers coordinated by sulphur-containing ligands and possess coordination sites that enable the binding and activation of molecular hydrogen.^13^Fig. 2 shows an example of the structure of a [NiFe]-hydrogenase. The active site of [NiFe]-hydrogenases consists of a binuclear Ni–Fe center. The iron atom is coordinated by three non-protein diatomic ligands, namely one carbonyl (CO) and two cyanide (CN^−^) ligands. The two metal ions are bridged by two cysteine residues coordinated in their thiolate form, which act as bridging ligands. In addition, the nickel center is coordinated by two terminal cysteine residues. This structural architecture plays a fundamental role in the activation and catalytic conversion of molecular hydrogen, but oxygen intolerance and chemical sensitivity of hydrogenase restrict its practical usage at a large scale.^11,13^

In this context, complexes capable of simultaneously reproducing the structural and functional features of the active site of the native enzyme are generally classified as synthetic analogues or biomimetic models. The literature reports a wide variety of model complexes developed based on hydrogenases for hydrogen generation.^14–18^ However, in this review, the focus was directed toward systems that, although inspired by these metalloenzymes, predominantly reproduce their catalytic activity without necessarily comprehensively mimicking the structural characteristics of the active site. Therefore, the discussion primarily considers complexes classified as functional models of hydrogenases.

The catalytic activity of these electrocatalysts is mostly associated with the combination of ligands in their coordination sphere with a well-defined metal centre and with the media in which they are inserted, for which various empirical modifications have been explored.^19,20^ Thus, an analysis of these bioinspired metal complexes regarding the advances they enable in HER electrocatalysis is necessary to understand their potential and push their application.

Studies on electrocatalysts for HER show theoretical and experimental data on how they work, as well as synthesis methods and results regarding their catalytic activity. However, it is essential to carry out a systematic review with a protocol, as it allows us to analyse the development of these materials for HER catalysis in a systemic way and find new horizons for their application. Thus, this study aimed to carry out a systematic review of bioinspired electrocatalysts in HER.

## 2. Methodology

### 2.1 Protocol and guidelines

This systematic review was conducted in accordance with the guidelines from the Preferred Reporting Items for Systematic Reviews and Meta-Analyses protocol (PRISMA). The process involved the identification, screening, eligibility and inclusion of studies on bioinspired metal complex electrocatalysts for HER. Thus, this research sought to answer in what ways bioinspired metal complexes have contributed to HER and what the electrocatalytic performance of these systems can be.

### 2.2 Search strategy

**Fig. 3:**
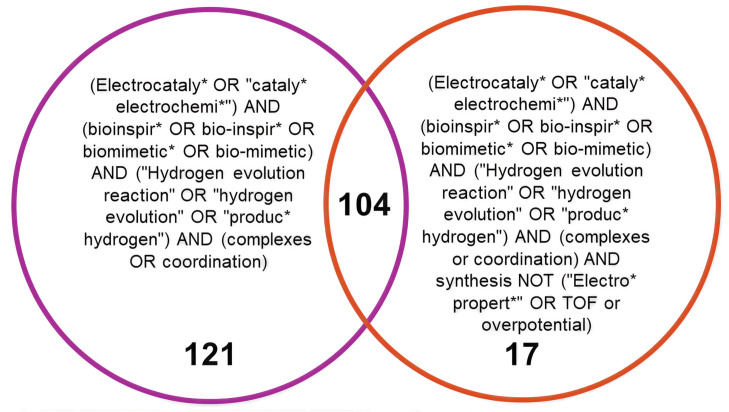
Set of search descriptors and search results.

The query was carried out on the Web of Science Core Collection database, in all editions, in February 2026, with no restrictions on year of publication. Descriptors were selected on topics, involving title, abstract and keywords, according to a previously defined strategy. Fig. 3 shows the search sets with the descriptors and Boolean operators used. The first search generically selected the studies of interest, while the second specified studies that dealt with synthesis while not addressing electrochemical properties and electrocatalytic performance. Thus, by using the NOT operator, this strategy excluded studies that only dealt with the synthesis of electrocatalysts. The use of asterisks made it possible to locate deriving terms and quotation marks located specific ones.

### 2.3 Selection of studies

**Fig. 4:**
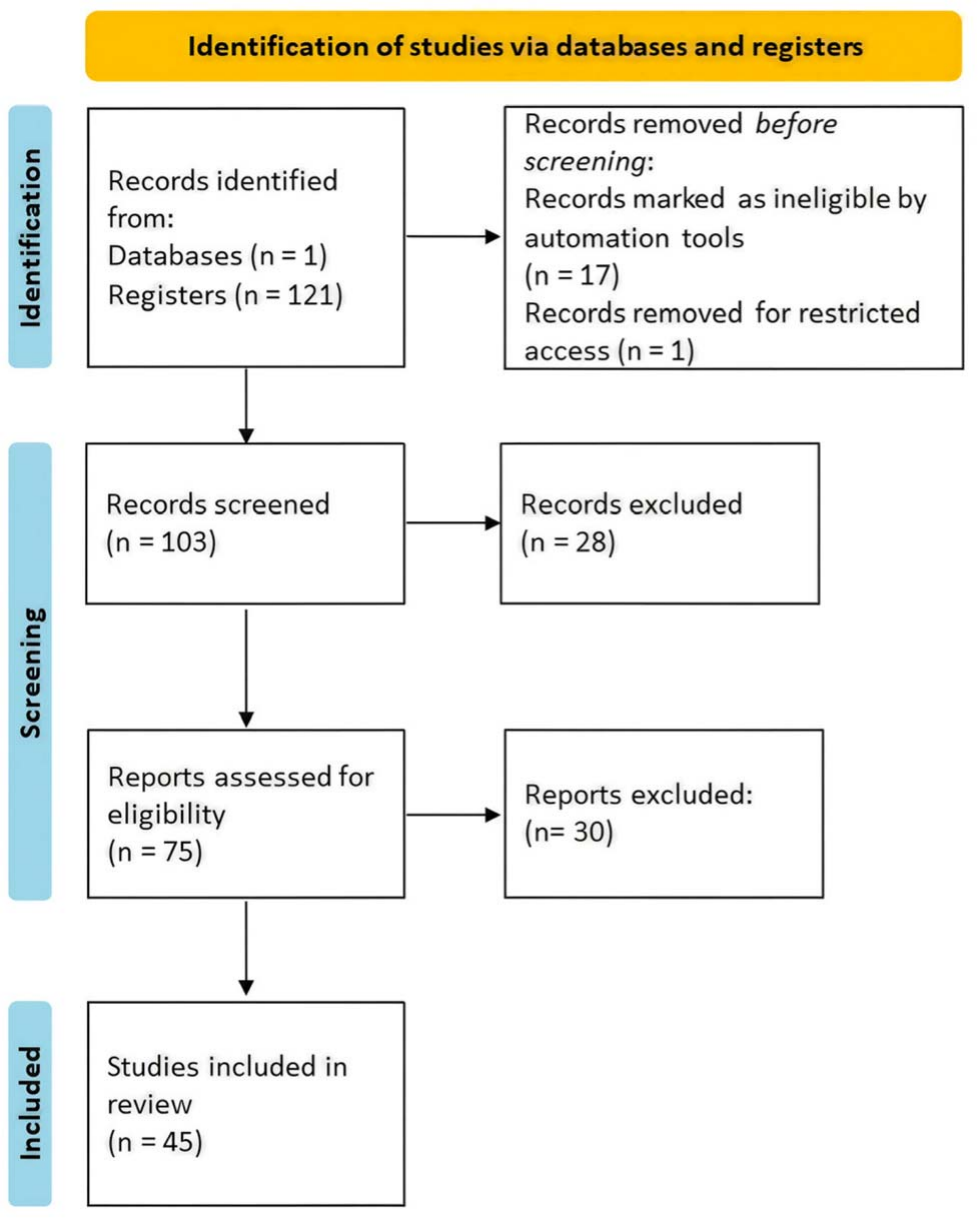
PRISMA flow diagram of the review search.^13^

The selection process was structured in four phases, according to the PRISMA model: identification, screening, eligibility and inclusion. During identification, records that had been identified through the Web of Science search strategy could be located. By screening these records, those that were not journal articles, but dealt with reviews, conferences and other types of publication, could be excluded, while only selecting studies written in English. Considering the remaining articles, the titles and abstracts were analysed for eligibility. Finally, applying the inclusion criteria, the articles were read in full and those that were outside the scope of the research were excluded. The relevant studies were included for a final quantitative analysis. The schematic of these PRISMA phases is shown in Fig. 4. The articles used in this review were stored electronically. The reference management software Zotero was used to consolidate and organize the references.

### 2.4 Eligibility criteria

#### 2.4.1 Inclusion criteria

(a) Articles addressing bioinspired metal complexes as electrocatalysts;

(b) Studies on the electrochemical properties of bioinspired electrocatalysts applied to HER;

(c) Manuscripts that present quantitative data on electrocatalytic performance, such as overpotential, turnover frequency (TOF) or electrochemical stability.

#### 2.4.2 Exclusion criteria

(a) Articles only focused on the synthesis, structural characterization of the complexes, or the study of theoretical mechanisms, without electrochemical analyses;

(b) Studies that do not address HER or that use non-bioinspired electrocatalysts and noble metals;

(c) Manuscripts with restricted access, which impeded a full analysis.

### 2.5 Data extraction

The data was extracted manually by reading the titles, abstracts and, then, the full text, collecting the following information: name of authors and year of publication; type of bioinspired metal complex; experimental conditions (electrolyte, solvent, pH, working electrode, reference electrode and counter electrode); electrochemical methods used (cyclic voltammetry, linear sweep voltammetry, among others) and electrocatalytic performance parameters (overpotential, turnover frequency, faradaic efficiency and stability).

## 3. Results and discussion

The records found using the descriptors applied on Web of Science were a total of 121 studies. After screening, 17 records were excluded because they were not journal articles and 1 was excluded because it was written in Mandarin. The titles and abstracts from the remaining 103 articles were analysed for eligibility, excluding 28 that did not meet the inclusion criteria. Finally, 75 articles were analysed in their entirety, causing the exclusion of 30 that were not directly related to the research, thus selecting 45 relevant articles for a final review with data extraction of HER catalysts. The articles included in the final review covered the period from 2007 to the present day, with an increasing number of papers published each year.

### 3.1 Types of metal complexes

The analysis of the articles included in this review revealed the structural and functional diversity of the catalytic architectures proposed for electrochemical applications in HER. Among the coordination compounds reported, some classes can be listed, such as mononuclear complexes, heterobimetallic complexes, intramolecular peptoids and even hybrid nanomaterials. Table 1 shows the types of complexes for each study.

**Table 1:** Types of complexes for each article

Article	Type of complex	Reference
01	[Mn(L)(CO)_3_]Br	21
02	Mono-nuclear Mn carbonyl complexes	22
03	(Et_4_N)_2_[Co^III^(qpdt)_2_]_2_	23
04	NiN_2_S_2_ and NOFeN_2_S_2_ metallodithiolates ligands	24
05	2Fe–2Se core supported by a b-diketiminato ligand	25
06	[FeFe] hydrogenase diiron model	26
07	Metallodithiolates|MN_2_S_2_·Fe(η^5^-C_5_R_5_)(CO)	27
08	Fe_2_S_2_(CO)_6_ core and a bridging ADT ligand	28
09	[Co(dimethylglyoxime)_2_(N_nucleobase derivative_)Cl]	29
10	Cobaloxime complex com isoniazid and hydrazide ligand	30
11	Vitamin B6-analog-coordinated cobaloxime	31
12	Cobalt-peptoid with terpy and bipy ligands – (CoTBE)	32
13	Bis(dithiolene)tungsten complexes	33
14	Molybdenum carbide-phosphide hybrid nanodots|Mo_2_C–MoP@CP	40
15	2Fe2S-f-CNT|[{(µ-SCH_2_)2N(C_6_H_4_CH_2_CO_2_R)}Fe_2_(CO)_6_]	41
16	Diiron dithiolate|[{(µ-SCH2)2N(C6H4CH2C(O)R)}Fe_2_(CO)_6_]	42
17	Diiron dithiolato CNT-X-ADT (X = N, C or O)|{(µ-SCH_2_)_2_N(CH_2_CO_2_C_6_H_4_CHO-p)}Fe_2_(CO)_6_	43
18	Carbon-nanotube-supported diiron monophosphine	44
19	Carbon-nanotube-supported diphosphine-chelate diiron	45
20	(Cu-doped g-C_3_N_4_) 0.31Cu–C_3_N_4_	46
21	Cobalt and nickel selenolate coordination polymers based on benzene-1,2,4,5-tetraselenolate (BTSe)	47
22	Ni(*Cys*-MPEG)_2_	48
23	Carbon-nanotube-supported Metalloprotein-cobaloxime-Azurin (CuAz)	49
24	Diiron complex mimicking the [FeFe]-hydrogenases and Metallopolymer (PDMAEMA)-*g*-[2Fe2S]	50
25	Cobalt-phosphonate coordination polymer|Co(CH_3_COO)_2_ 3moppH_2_	51
26	Dimeric nickel(ii) supported by tridentate NS2 ligands	52
27	Nickel(ii) diselenolate [Ni(bds)(dppf)]	53
28	[Ni(xbsms)Mn(CO)_3_(H_2_O)]^+^	54
29	β-Fluorinated tunichlorin mimics	55
30	Diiron complex Fe_2_(S_2_C_3_H_6_)(CO)_6_	56
31	[Cl–Cu-LN_2_S_2_]ClO_4_	57
32	(*CoTBImPc*) + CNT and cobalt tetra[4-[2-(1*H*-benzimidazol-2-yl)phenoxy]]-phthalocyanine	62
33	Cobalt diimine-dioxime immobilized onto carbon nanotube	63
34	Cobalt and nickel diimine-dioxime	64
35	Thiolate-bridged dicobalt hydride|[Cp*Co(µ-SR)_2_CoCp*]	65
36	Iron-carbonyl|{CpFe(CO)2}	66
37	Pentacoordinate mononuclear iron carbonyls	67
38	µ-Oxo diiron|[Fe^III^–(µ-O)–Fe^III^]	68
39	Binuclear iron complexes with CO and phosphine ligands	69
40	Sac catalysts models	70
41	Sac/CoP composite with cobalt porphyrin	71
42	Covalent and non-covalent 2Fe2S/CNT hybrid	59
43	Bioinspired ruthenium–Porphyrin	58
44	Metal–NHC complexes featuring bioinspired [Fe_2_(µ-adt)(CO)_6_] units	60
45	Bioinspired [NiFe]-hydrogenase complex [L^N2S2^Ni^II^Fe^II^Cp(CO)]^+^	61

**Fig. 5:**
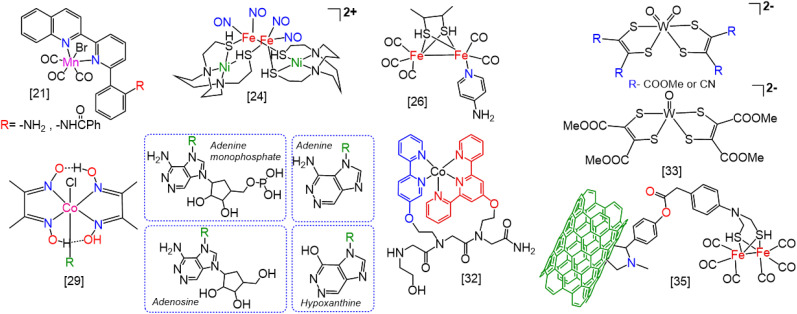
Complex structures used as HER electrocatalysts. *The reference article number is shown in brackets.

Mononuclear complexes such as [Mn(mesbpy)(CO)_3_(MeCN)]^+^ and [Mn(CO)_3_(PPh_(2)_Py)] stand out for their structural simplicity and functionality in the electrocatalysis of HER with manganese as the only active metal centre^21,22^ (Fig. 5). The presence of the mesbpy ligand (4,4′-dimethyl-2,2′-bipyridine) and PPh_2_Py (diphenyl-2-pyridylphosphine) contributes to the steric and electronic stabilization of the active site. The addition of more ligands to the coordination sphere increases the catalytic capacity, which are responsible for stabilising and catalysing proton reduction. These systems are widely used in CO_2_ reduction reactions and the transformation of small molecules. Similarly, [Co^III^(qpdt)_2_]_2_ has the only cobalt(iii) active site coordinated by the qpdt ligand, exerting catalytic activity on HER.^23^ Thus, biomimetic inspiration aims to mimic hydrogenase enzymes, optimizing selectivity and kinetics for hydrogen evolution processes.

Biomimetic models of [FeFe] hydrogenases, such as the ((µ-dmedt)Fe_2_CO)_6_ complexes, are recurrent in the literature. In this sense, Ghosh *et al.* synthesized a matrix of complexes involving nickel and iron, forming bimetallic arrangements that allow cooperative electronic interaction between both metals.^24^ Three variants were tested: [Ni–Fe]^+^, [Fe–Fe]^+^ and [Ni–Fe′]^+^, demonstrating how the variation in structural arrangement directly influences catalytic activity and efficiency. Furthermore, the incorporation of dithiolate ligands such as (dmedt = 2,3-butanediol) allows one to replicate the geometry and function of the active centres for HER.^25–27^ In addition, azadithiolate (adt) and oxadithiolate (odt) bridged complexes have been explored to modulate the electronic density of the metal and optimize charge transfer.^28^

In general, cobaloximes continue to be a focus of intense study for their activity in HER. Systems functionalised with nucleobase derivatives, isoniazid or vitamin B6 analogues, have shown catalytic performance modulated by the introduced supramolecular interactions^29–31^ (Fig. 5). Thus, adding peptoid-type ligands also enhances the stability and selectivity of cobalt complexes. Pahar and Maayan reported the synthesis of an intramolecular cobalt complex in which the bond between terpyridine and bipyridine units is mediated by a peptoid, forming a rigidly defined intramolecular active site.^32^ This configuration seeks to mimic the precise organization of metal cofactors in enzymes.

Complexes with metals such as molybdenum (Mo), tungsten (W) and nickel (Ni) have gained prominence as well. Tungsten bis–dithiolene complexes show excellent ability to mimic redox enzymes with high stability^33^ (Fig. 5). On the other hand, hybrids such as Mo_2_C–MoP@CP represent the intersection between molecular catalysis and nanometric materials. Since most enzyme-bioinspired molecular complexes exhibit low electrical conductivity, conductive solid materials, such as carbon nanotubes (CNTs) and other hybrids, are developed and applied in the HER targeting industrial viability.^34^ These solid systems offer favorable pathways for efficient charge transfer and electrical conductance, while also providing an increased surface area that allows for a higher catalyst loading on the electrode.^35–38^ Additionally, heterogeneous catalysts are more stable and exhibit more facile recovery than their homogeneous counterparts.^39^ An *et al.* developed molybdenum carbide-phosphide hybrid nanodots immobilized on commercial carbon paper.^40^ This synthesis was mediated by polydopamine, conferring biomimetic characteristics and promoting efficient anchoring of the nanodots. This complex structure, thus, provides a large surface area and excellent electrical conductivity.

Various complexes have been incorporated into supports such as carbon nanotubes (CNTs) or doped graphite, creating high-performance heterogeneous systems^41–46^ (Fig. 5). Immobilization on CNTs, for example, improves accessibility to the catalytic site and the operational stability of the metal complex in HER. On the other hand, polymer systems, such as the polymeric coordination of cobalt with phosphonate ligands, illustrate the growing trend to integrate catalysts into robust structural matrices.^47,48^ These materials offer the advantage of reusability and easy handling.

The architectural diversity demonstrates that modulating the structure of the complex is a key strategy for improving catalytic activity. Biomimetic complexes offer advantages in terms of selectivity, but often lack long-term stability. On the other hand, nanometric and hybrid architectures combine a large surface area with improved stability, and are promising for large-scale practical applications. Heterobimetallic complexes stand out due to the possibility of electronic synergies, but require precise synthetic control to avoid unwanted processes such as ligand dissociation.

### 3.2 Experimental conditions

Electrochemical conditions as determinants of catalytic efficiency and selectivity varied between studies, reflecting the diversity of catalytic systems. The factors that have notably influenced the catalytic activity of HER involve pH, solvent and electrolytes employed, as well as the electrode types involved in the experiments.

**Fig. 6:**
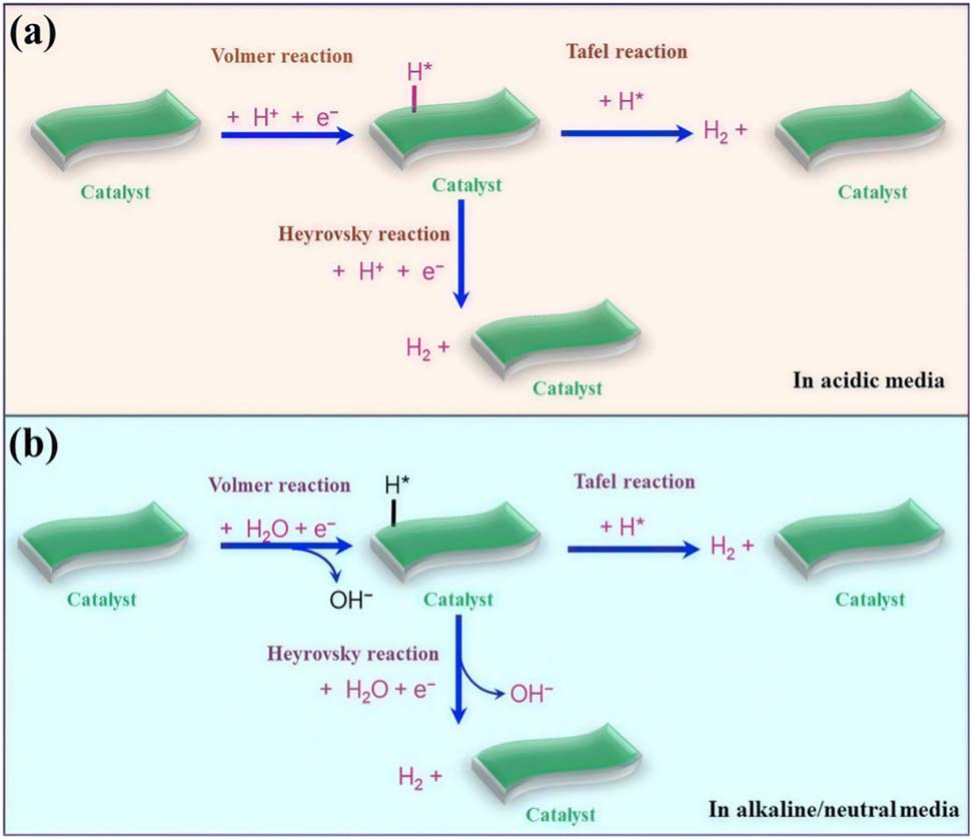
Illustrative overview of HER pathways occurring in different electrolytic environments: (a) acidic medium and (b) basic/neutral medium. This figure has been adapted from ref. 8 with permission from Tran *et al.*, copyright 2026.

Aqueous media (Fig. 6) that provide neutral conditions are indicated for environmental reasons, so efforts made to carry HER operations at a pH of approximately 7.0 have been described.^32,42,43,45,49–52^ The search for systems capable of operating efficiently in neutral conditions has led to electrochemical tests in reaction media with different pH: acidic, alkaline and neutral.^41,47^ This pH variation is generally determined by the electrolyte and solvent used in the experiments.

Because of proton transfer, these systems tend to have better catalytic performance in acidic conditions. From this perspective, other studies report experimental tests only at acidic and neutral pH in the range from 4 to 7.^29–31,49^

The choice of pH is intrinsically linked to the stability of the complex and to its catalytic efficiency: while acidic media favour the evolution of H_2_, alkaline media may be more compatible with materials based on less noble transition metals. Furthermore, pH influences not only the thermodynamics of the reaction, but also the stability of the complex: many metal complexes undergo rapid deactivation in highly acidic or alkaline environments, requiring an excellent combination in the experimental media.

When a combination of experimental conditions is established, electrolytes play an important role, being fundamental in determining some factors such as medium pH and polarity. Electrolytes have basically ranged from organic salts to strong bases and buffer solutions, such as tetraalkylammonium [*n*-Bu_4_] [PF_6_]/[BF_4_] with the alkyl group being butyl or ethyl in hexafluorophosphate or tetrafluoroborate.^21,22,27,53–56^ These salts are used in concentrations of 0.1 mol L^−1^, usually associated with solvents due to their electrochemical window. As with aqueous media, there is a variety of soluble inorganic salts such as alkali metal halogens that are considered excellent electrolytes.^25,40,57,58^

Phosphate buffer electrolytes are also used, as they are able to make the medium approximately neutral for HER electrocatalysis.^32,42,43,50^ Thus, the choice of electrolytes aims to ensure good ionic conduction, complex stability, electrode compatibility and the formation of ionic pairs or micellar aggregates with electroactive species. It is an important decision as it can impose positive or negative voltage limits due to the redox properties of the electrolyte.

Electrochemical catalysts for HER involve metal complexes with low solubility in water, thus requiring suitable solvents for their correct functionalization. Regarding solvents, most studies have used aphotic organic solvents such as acetonitrile (ACN), *N*,*N*′-dimethylformamide (DMF) and dichloromethane (DCM), which are widely used due to their wide electrochemical window and good solubility for metal complexes.^21,22,24,53–55,57,59–61^ The predominant use of organic solvents, although effective for dissolving complexes, raises environmental and safety concerns, indicating a growing need for aqueous systems or green solvents.

When determining the catalytic performance parameters, the electrode type directly influences the potentials observed and the interpretation of the catalytic activity. The previously mentioned studies follow the standard electrochemical cell model for HER with three-electrode systems, *i.e.*, working electrode, reference electrode and counter electrode (Table 2).

**Table 2:** Summary of catalysts, electrochemical experimental conditions, TOF, faradaic efficiency and overpotential of the articles included in the systematic review from the last 5 years (2021 to 2026)^a^

Catalyst	Electrochemistry			TOF (s^−1^)	Faradaic efficiency (%)	Overpotential (mV)	Ref
	Working electrode	Electrolyte	Solvent				
[Mn(L)(CO)_3_]Br	GC	[C_6_H_5_NH_3_][BF_4_]	CH_3_CN and CH_3_CN/DMF	>10 000	—	700	21
Mono-nuclear Mn carbonyl complexes	GC	[N(*n*-Bu_4_)][PF_6_]	CH_3_CN	610–615	—	1010–1020	22
[Co(dimethylglyoxime)_2_(N_nucleobase derivative_)Cl]	GC	Na_2_SO_4_	Aqueous	∼13 000	83–92	< 400	29
Cobaloxime complex com isoniazid and hydrazide ligand	GC	Na_2_SO_4_	Aqueous	794–3630	86–89	466–498	30
Vitamin B6-analog-coordinated cobaloxime	GC	Na_2_SO_4_	Aqueous	24–1370	> 83	321	31
2Fe2S-f-CNT|[{(*µ*-SCH_2_)2N(C_6_H_4_CH_2_CO_2_R)}Fe_2_(CO)_6_]	Graphite	KCl	Aqueous	0.07716	—	510–900	41
Diiron dithiolate|[{(µ-SCH2)2N(C6H4CH2C(O)R)}Fe_2_(CO)_6_]	Catalyst-loaded GC	KCl	Aqueous	—	—	1220	42
Diiron dithiolato CNT-X-ADT (X = N, C or O)|{(µ-SCH_2_)_2_N(CH_2_CO_2_C_6_H_4_CHO-p)}Fe_2_(CO)_6_	Hybrid-modified GC	KCl	CH_3_CN	0.175	—	880–1000	43
Carbon-nanotube-supported diiron monophosphine	Catalyst-modified GC	KCl	Aqueous	—	92–97	457	44
Carbon-nanotube-supported diphosphine-chelate diiron	Catalyst-modified GC	KCl	Aqueous	0.9	—	100	45
Ni(*Cys*-MPEG)_2_	Graphite	Na_2_SO_4_	Aqueous	8–144	∼90	450	48
Carbon-nanotube-supported Metalloprotein-cobaloxime-Azurin (CuAz)	GC	TBAPF_6_	Aqueous and organic	2 × 10^5^	97–100	390	49
Cobalt-phosphonate coordination polymer|Co(CH_3_COO)_2_ 3moppH_2_	GC	KCl	Aqueous	0.008–0.95	98–100	490	51
β-Fluorinated tunichlorin mimics	GC	[*n*Bu_4_]PF_6_	CH_3_CN	270–24 000	100	1100	55
[Cl–Cu-LN_2_S_2_]ClO_4_	GC	KCl	Aqueous	241.75	94	800	57
µ-Oxo diiron|[Fe^III^–(µ-O)–Fe^III^]	GC	TBAPF_6_	CH_3_CN	1.4 × 10^5^–17 × 10^5^	91–95	500–1050	68
Covalent and non-covalent 2Fe2S/CNT hybrid	GC	[*n*Bu_4_]PF_6_	CH_3_CN	0.533–0.049	—	1070–1220	59
Bioinspired ruthenium–porphyrin	Graphite	KOH	Aqueous	4.73–9.02	—	42–348	58
Metal–NHC complexes featuring bioinspired [Fe_2_(µ-adt)(CO)_6_] units	GC	[*n*Bu_4_]PF_6_	CH_2_Cl_2_	0.6–5	—	1040–1070	60
Bioinspired [NiFe]-hydrogenase complex [L^N2S2^Ni^II^Fe^II^Cp(CO)]^+^	GC	[*n*Bu_4_]PF_6_	CH_3_CN	0.8–5000	70	500	61

a Experimental conditions: a three-electrode system was used in all cases, featuring a platinum counter electrode. TOF and faradaic efficiency fields in blank were not provided by the original authors. [C_6_H_5_NH_3_][BF_4_] – anilinium tetrafluoroborate; [N(*n*-Bu_4_)][PF_6_] – tetrabutylammonium hexafluorophosphate; TBAPF_6_ – Tetrabutylammonium hexafluorophosphate.

The types of electrodes used in those studies were relatively homogeneous, with the working electrode being mostly the glassy carbon electrode (GCE), especially in the (1 mm to 3 mm) disk form.^21,22,30,32,53,57,60–62^ The use of GCE is justified by its electrochemical inertness, low parasitic overpotential and good reproducibility. For materials in heterogeneous electrochemicals, CNTs anchored with the complex were supported directly on the working electrode, maximizing the electrode-catalyst interaction.^40–42,44,46,51^

**Fig. 7:**
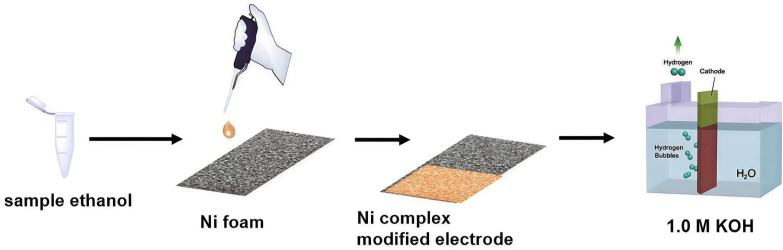
Schematic diagram showing how the catalyst can be loaded onto a metal foam. To prepare the working electrode, the catalyst powders were ultrasonically dispersed in volatile solvent, then the suspension was dropped onto a metal foam electrode, such as Ni. The electrode is dried at room temperature and it is ready to use as the working electrode. This figure has been adapted from ref. 55 with permission from Xue *et al.*, copyright 2024.

When modified with nanomaterials, an electrode influences the obtained current density and the contact resistance. A simple method to increase a catalyst's active surface area in electrochemistry is the use of modified metal foams. Xue *et al.* applied a dispersion with the catalyst (a series of Ni complexes) to a nickel foam in order to build the working electrode (Fig. 7).^55^ Regarding the reference electrode, several systems have used more traditional references such as Ag/AgCl or Hg/HgO.^30,32,40,51,57,62^ The choice of reference electrode is essential to ensure the reliability of the potentials recorded. In some studies, in addition to the reference electrode, the ferrocene/ferrocenium (Fc^0^/^+^) redox pair is used as an internal standard to report the collected potentials, with the most appropriate being reporting in relation to the normal electrode (ENH) or standard electrode (EPH).^21,24,26,54,56^ Finally, the counter electrode, when mentioned in the articles, was typically platinum wire or a carbon rod, due to their stability and conductivity.^22,27,32,40,41,46,53^ The electrochemical cell configurations for the application of methods capable of quantifying catalytic performance in HER are therefore reported.

Hence, these methodological variations highlight the importance of standardizing or, at least, reporting the experimental conditions in detail, to enable direct comparisons between the different catalytic systems studied.

### 3.3 Electrochemical methods and techniques

**Fig. 8:**
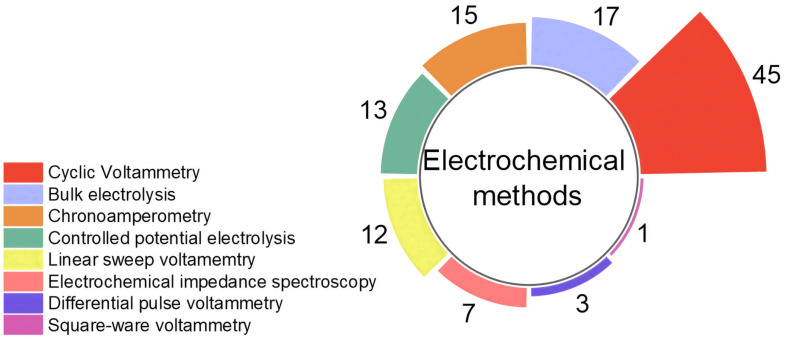
Electrochemical methods used in the papers. More than one method might be used in the same study.

Electrochemical methods are used to quantify the efficiency of the catalysts used in HER. In this review, all the explored studies applied multiple electrochemical methods to characterize the catalytic activity and stability of their systems, as Fig. 8 shows.

**Fig. 9:**
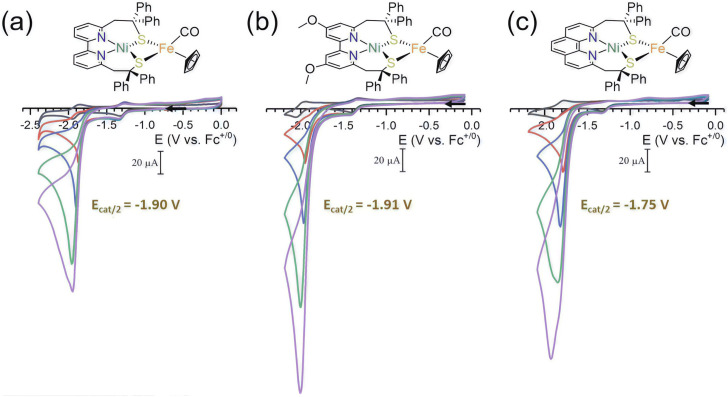
CVs of 0.20 mM of LNiFe (a), L^OMe^NiFe (b), and L^Phen^NiFe (c), before (dark grey) and after addition of various amounts of Et_3_NHBF_4_ in 0.1 M NBu_4_PF_6_/MeCN solution at 100 mV s^−1^: 5 equiv (red), 10 equiv (blue), 20 equiv (green), 30 equiv (purple), and 40 equiv (yellow).^65^ This figure has been adapted from ref. 65 with permission from Wang *et al.*, copyright 2020.

One of the main techniques for quantifying electrocatalytic performance parameters, such as overpotential and current density, is cyclic voltammetry (CV)^21–33,40–71^. This universally used method has made it possible to evaluate complex redox processes, identify oxidation/reduction potentials and make inferences about the mechanism of catalytic action. Lalaoui *et al.* evaluated the influence of the amount of Et_3_NHBF_4_ as a proton source for LNiFe(a), L^OMe^NiFe(b), and L^Phen^NiFe(c) used in CV, evaluating the half-wave potential *E*_cat/2_ (Fig. 9), which allowed them to establish that all complexes can reduce protons in organic media.^61^

Although CV has been essential in identifying the starting potentials of HER reactions, there is a competing technique called linear sweep voltammetry (LSV), which performs a linear sweep of the potential and is used in several studies.^21,40–45,49,54,58,60,61,63,64^ The LSV technique was used in the studies along with CV, making it possible to compare the potentials found in HER catalysis.

In electrolysis approaches, controlled potential electrolysis (CPE) and chronopotentiometry techniques are important to determine how stable the catalysts are during the HER process. These techniques have been applied in some studies, which provided information on the operational stability of the catalysts over long periods of electrolysis.^21,24,25,32,40–43,47,54,56,58,59,62^ In this perspective, An *et al.* demonstrated the functional stability of their Mo_2_C–MoP nano catalyst for more than 120 h.^40^ Thus, regarding the turnover frequency and stability of electrocatalysts, these are important analysis techniques.

From the perspective of investigating the electrical parameters of metal complex catalysts for HER, electrochemical impedance spectroscopy (EIS) is an efficient method. The reported approaches to the application of EIS have shown its relevance in the analysis of factors such as charge resistance (*R*_ct_) and double layer capacitance (*C*_dl_).^40,43–45,58,59,63^ The application of this method has been important in the analysis of systems based on hybrid materials or those supported by CNTs.

The predominance of CV reflects its ease of application and the wealth of information obtained, but it also highlights a limitation: the excessive dependence on this technique, which can mask aspects related to stability and durability over long operations. Theoretical studies on the application of density functional theory (DFT) have been able to elucidate complex catalytic mechanisms.^21,22,24,33,53,55,57,61^ Thus, the combination of these techniques provides a comprehensive insight into catalytic efficiency, stability and mechanisms.

### 3.4 Catalytic performance parameters

Quantitative analyses of the catalytic performance of electrocatalysts for HER are fundamental for determining some important factors related to the efficiency of these materials. In order to optimize the activity of electrocatalysts, some parameters such as overpotential, turnover frequency (TOF), faradaic efficiency and long-term stability need to be known (Table 2). Thus, these data were analysed to understand the catalytic performance in HER.

The overpotential is one of the main indicators of a catalyst's energy efficiency, reflecting the difference between the ideal thermodynamic potential and the one actually required to initiate the electrochemical reaction. The overpotential was calculated using eqn (1):^30^

1 Overpotential (OP) = *E*_cat/2_ − *E*_H^+^/H_2__

Among the systems analysed, a catalyst based on metal-doped polydopamine stood out with a low overpotential of 147 mV, suggesting high efficiency in HER activation.^40^ A competing system with an overpotential value of approximately 180 mV was reported for a synthetic complex inspired by the active site of azadithiolate-bridged hydrogenase.^28^

**Fig. 10:**
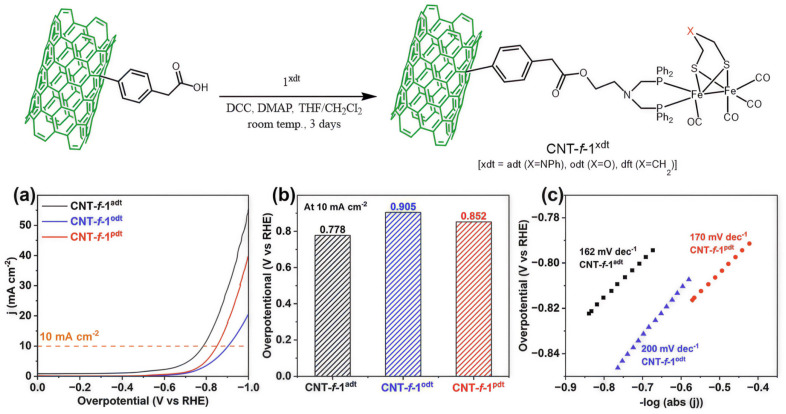
Synthesis of the CNT-attached hybrid catalysts CNT-f-1^xdt^ (xdt = adt, odt, and pdt) and comparison for electrochemical HER properties of hybrid catalysts CNT-f-1^xdt^ (xdt = adt, odt, and pdt) which were respectively loaded onto GC electrodes with a loading of 0.71 mg cm^−2^ in 0.05 M PBS solution (pH 7.4). (a) LSV curves; (b) the overpotentials at 10 mA cm^−2^; (c) Tafel slope. This figure has been adapted from ref. 39 with permission from Wang *et al.*, copyright 2025.

Zhao *et al.* evaluated three CNTs functionalized with [FeFe]-hydrogenase-inspired complexes: CNT-*f*-1^adt^, CNT-*f*-^odt^, and CNT-*f*-^pdt^. The authors discuss the overpotential (Fig. 10a–c), demonstrating a faster kinetic process catalysed by CNT-*f*-1^adt^, which is attributed to the structure of the chelate compound, as the presence of the nitrogen atom in the dithiolate bridge facilitates proton transport, enhancing its efficiency.^45^ On the other hand, mononuclear cobalt and manganese systems showed a relatively high overpotential of 550 mV and 700 mV, respectively, indicating the need for structural or electronic optimizations.^21,23^ This contrast highlights the importance of the coordination of the central metal, the structure of the ligand and the electronic conductivity of the medium. On the other hand, the study by Ghosh *et al.* is notable for their investigation into different heterobimetallic pairs.^24^ The overvoltage values ranged from 660 mV [Fe–Fe] to 711 mV [Ni–Fe], with a slight superiority of the system based exclusively on iron. This variation suggests that the electronic and geometric nature of the metal pair directly influences the activation energy of the reaction.

The TOF represents the number of molecules catalysed per unit of time per active site, reflecting the intrinsic activity of the catalyst. However, not all studies report it, making comparative evaluations difficult (Table 2). TOF can be calculated in several ways, but all equations take into account the number of moles of H_2_ produced, the surface loading measured by CV, or the current obtained from LSV, for example.^49,58,68^

The mononuclear manganese catalyst stands out regarding TOF, at 5.41 × 10^3^ s^−1^, indicating that, despite the high overpotential, the active site has high individual catalytic efficiency.^21^ In contrast, an intramolecular cobalt complex system, in which the bond between terpyridine and bipyridine units is mediated by a peptoid, with a TOF of 44 s^−1^, shows more modest performance, but still significant for systems based on peptoid structures, which are relatively new in the field.

In turn, Ghosh *et al.* reported varying TOFs: 39.7 s^−1^ [Ni–Fe] and 26.7 s^−1^ [Fe–Fe], which complements their overpotential analysis.^24^ Although [Fe–Fe] requires less overvoltage, its TOF is lower, indicating a possible trade-off between energy efficiency and intrinsic activity.

**Fig. 11:**
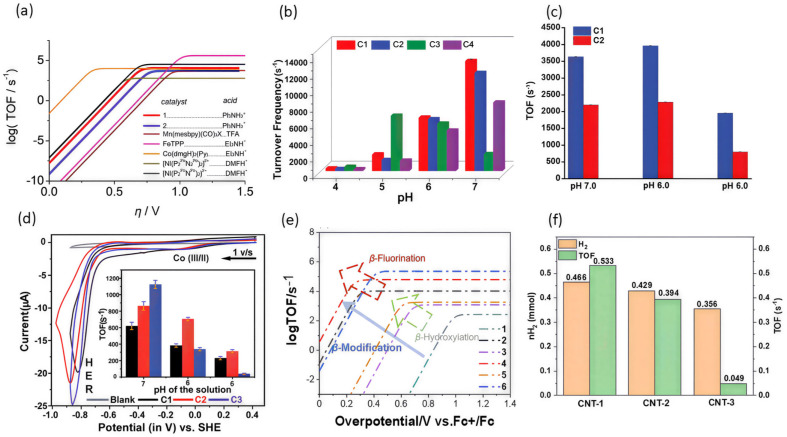
(a) Catalytic Tafel plots relating TOF and driving force of H_2_ evolution, and benchmarking of the performances of 1 and 2 (this work) with previously reported H_2_-evolving electrocatalysts: 1-[Mn(L1)(CO)_3_]Br; 2-[Mn(L2)(CO)_3_]Br; Mn(mesbpy)(CO)_3_X, FeTPP, Co(dmgH)_2_(py), [Ni(P_2_^Ph^N_2_^Ph^)_2_]^2+^, and [Ni(P_2_^Ph^N_2_^Ph^)_2_]^2+^.^21^ (b) Comparative electrocatalytic H_2_ production rate (TOF) and variable pH conditions, for Co(dimethylglyoxime)_2_ClL_1-4_ (L1 = Adenosine monophosphate, L2 = Adenosine, L3 = Adenine, L4 = Hypoxanthine).^29^ (c) Comparative electrocatalytic H_2_ production rate (TOF) and variable pH conditions, for Co(dimethylglyoxime)_2_ClR_1–2_ (R1 = Isoniaszid, R2 = Nicotinic hydrazide).^30^ (d) Comparative cyclic voltammogram data recorded for complexes C1 (black line), C2 (red line), and C3 (blue line), and a blank (grey line) at pH 7.0 under anaerobic conditions. The inset highlights the comparative electro catalytic H_2_ production TOF values calculated for cobaloxime cores with 3-hydroxy2-methylpyridine (C1), pyridoxamine(C2), andpyridoxal(C3) at pH 5.0 to 7.0^31^. (e) Catalytic Tafel plots to benchmarking the performances of 1 to 6, where β-fluorinated tunichlorin mimics.^48^ (f) Comparative electrocatalytic H_2_ production and TOF, for CNT-1, CNT-2, CNT-3.^62^ This figure has been adapted from ref. 21, 29, 30, 31, 48, 62. Copyright 2021–2025, the Author(s).

Fig. 11 summarizes several correlations used to evaluate the efficiency of complexes for H_2_ generation. In Fig. 11(a), the authors compare their reported catalysts with the literature; the TOF is very similar between this class of compounds and nickel complexes with amine groups in their structure, demonstrating a promising chemical environment for HER in all cases.^21^Fig. 11(b)–(d) evaluate the TOF in relation to pH for a series of Co(dimethylglyoxime)_2_ClL complexes, where L varies with different nucleotides. In all studies, a higher TOF is observed at pH 7, proving to be an important parameter in H_2_ generation, as previously discussed in this review.^29–31^ In Fig. 11(e), the authors evaluated a series of β-fluorinated tunichlorin mimics regarding overpotential, comparing them with β-modification and β-hydroxylation; the compounds modified by the authors showed a significant increase in HER rates.^55^ Finally, Fig. 11(f) displays the complexes anchored onto CNTs, with the authors correlating the amount of H_2_ generated with the TOF, demonstrating a direct relationship between calculated TOF values and H_2_ production.^59^

Faradaic efficiency is an important parameter for evaluating the selectivity of the catalyst for the desired product, which is hydrogen in the case of HER (Table 2). It can be calculated according to eqn (2):^21^

2 Faradaic yield product (%) = 100 × *n*_prod_/(*Q*/*F*/2)

Where *F* is the Faraday constant (C mol^−1^), *n*_prod_ (mol) is the amount of H_2_ in the headspace determined by GC, and *Q* (C) is the charged passed during electrolysis.^21^

Zamader *et al.* studied diiron complexes with similar chemical environments, one of which was anchored to CNTs, and both exhibited a faradaic efficiency between 97% and 100%. This highlights the crucial role that this dinuclear metallic structure, featuring π-acid ligands, plays in proton transfer for H_2_ generation.^49,51^

Mononuclear manganese and cobalt complex systems were 95% and 90% efficient, respectively.^21,23^ In comparison, the peptoidal metal complex catalyst reached 92%.^32^ These high values indicate low by-product production and good selectivity. In contrast, the catalysts by Ghosh *et al.* showed intermediate values of 68% [Ni–Fe] and 58% [Fe–Fe].^24^ This suggests that the presence of nickel can improve the selectivity of the process. However, the absence of faradaic efficiency data in some studies^25,40,41,46,51,57^ reinforces the need for more complete characterization in future works on new catalysts.

Stability is essential for practical applications. Catalyst stability is determined by the time it retains catalytic activity without degrading, while maintaining structural integrity during the reaction. The study on the Mo_2_C–MoP nano catalyst stood out by demonstrating stability for more than 120 h, indicating a robust and durable system.^40^ In this sense, complexes that mimic hydrogenases with diiron nuclei also showed good stability for 18 h.^25^ Thus, these catalysts had a curious behaviour of gradual improvement in performance, possibly due to surface restructuring or electrochemical activation.

From this perspective, Pahar and Maayan's peptoidal catalyst showed consistent performance for at least 10 h.^32^ This shows sufficient stability for short-to-medium-term applications. Ghosh *et al.* reported moderate stability, however without quantification, which limits conclusions about its applicability.^24^ This quantification would allow us to reflect more broadly on the electrochemical stability of some of the main catalysts over medium and long periods of operation.

The integrated analysis shows that the most promising catalysts are those that balance good energy efficiency (low overpotential), high intrinsic activity (TOF), high selectivity (faradaic efficiency) and robustness (stability), all summarized in Table 2. However, some studies still lack more comprehensive metrics, which makes it difficult to make direct comparisons and build a solid basis for the rational development of new catalytic materials.

## 4. Conclusions and outlook

The studies show that the evolution of bioinspired metal complexes is moving towards structural and functional sophistication. The diversity of metals and ligands reflects a search for systems that are increasingly efficient, selective and adaptable to real contexts of application in catalysis and energy. Regarding experimental conditions, the influence of pH on catalytic reactions is notable, affecting both the stability of the complexes and the proton transfer mechanisms. The use of organic solvents is justified by the solubility of the complexes, but poses challenges in terms of sustainability and biocompatibility. The choice of electrodes directly influences the potentials observed and the interpretation of catalytic activity. However, some studies fail to mention the counter electrode, which compromises reproducibility and interlaboratory comparisons.

In catalytic performance analysis techniques, CV stands out as a primary diagnostic method in molecular electrocatalysis. Greater inclusion of EIS and other advanced spectrum electrochemical techniques could broaden the understanding of charge transport mechanisms and resistance to deactivation. The data analysed demonstrate significant advances in the engineering of metal catalysts for electrochemical applications, highlighting the need for greater standardization of reported parameters, as well as the expansion of long-term stability studies and integration of theoretical and experimental studies. The diversity of approaches and results stresses the complexity of the field and the need for multidisciplinary strategies for the development of highly efficient, selective and stable catalysts.

## Conflicts of interest

There are no conflicts to declare.

## Data Availability

Article review: bioinspired metal complexes as electrocatalysts for hydrogen evolution: A systematic review. No primary research results, software or code have been included and no new data were generated or analysed as part of this review. Data citation are included in bibliographic references, as recommended by the RSC instructions.
